# Thrombozytenaggregationshemmer (Update 2023)

**DOI:** 10.1007/s00508-023-02168-6

**Published:** 2023-04-20

**Authors:** Thomas C. Wascher, Thomas M. Stulnig, Christoph H. Saely, Lars Stechemesser, Ausschuss Leitlinien

**Affiliations:** 1 1. Medizinische Abteilung, Hanuschkrankenhaus, Wien, Österreich; 2 3. Medizinische Abteilung und Karl Landsteiner Institut für Stoffwechselerkrankungen und Nephrologie, Klinik Hietzing, Wien, Österreich; 3 Abteilung für Innere Medizin I, Akademisches Lehrkrankenhaus Feldkirch, VIVIT Institut, Feldkirch, Österreich; 4 Private Universität im Fürstentum Liechtenstein, Triesen, Liechtenstein; 5 Universitätsklinik für Innere Medizin I, Paracelsus Medizinische Privatuniversität, Müllner Hauptstraße 48, 5020 Salzburg, Österreich

**Keywords:** Thrombozyten, Aggregationshemmer, Akute Atherothrombose, Diabetes mellitus, Platelets anti platelet drugs, Acute atherothrombosis, Diabetes mellitus

## Abstract

Akute atherothrombotische Komplikationen tragen, im Rahmen der beschleunigten Atherosklerose, zur kardiovaskulären Morbidität und Mortalität von Menschen mit Diabetes bei. Die Hemmung der Thrombozytenaggregation kann das Risiko für das Auftreten akuter atherothrombotischer Komplikationen reduzieren. Der vorliegende Artikel stellt die Behandlungsvorschläge der österreichischen Diabetesgesellschaft zum Einsatz von Thrombozytenaggregationshemmern dar.

## Abstract (en)

Acute thrombotic complications as a key feature of accelerated atherothrombotic disease typically precipitate cardiovascular events and therefore strongly contribute to cardiovascular morbidity and mortality in patients with diabetes. Inhibition of platelet aggregation can reduce the risk for acute atherothrombosis. The present article represents the recommendations of the Austrian Diabetes Association for the use of antiplatelet drugs in patients with diabetes according to current scientific evidence.

## Grundsatz Statement

Eine medikamentöse Hemmung der Thrombozytenaggregation reduziert kardiovaskuläre Morbidität und Mortalität bei Patienten mit manifester kardiovaskulärer Erkrankung sowie mit hohem kardiovaskulärem Risiko. Ebenso jedoch erhöht diese Therapie das Risiko für Blutungskomplikationen.

## Indikation zur Therapie

Als Basis der Therapieindikationen dienen die ESC/EASD Leitlinien zu Diabetes und Prädiabetes 2019 [1]. Das kardiovaskuläre Risiko von Menschen mit Diabetes wird dabei wie folgt kategorisiert:

- Sehr hohes Risiko
  - Diabetes mit kardiovaskulärer Erkrankung
  - Diabetes mit Endorganschäden (Albuminurie, Retinopathie, Neuropathie)
  - Diabetes mit ≥ 3 weiteren Risikofaktoren (Alter (Männer > 50 Jahre, Frauen > 60 Jahre), Hypertonie, Hyperlipidämie, Rauchen, Adipositas)
  - Typ 1 Diabetes mit früher Manifestation und > 20 Jahren Krankheitsdauer.
- Hohes Risiko
  - Diabetes ohne Endorganschäden aber einer Krankheitsdauer von ≥ 10 Jahren oder zumindest mit einem weiteren Risikofaktor.
- Mittleres Risiko
  - Junge Patienten (Typ 1 Diabetes < 35 Jahre, Typ 2 Diabetes < 50 Jahre) mit Diabetes-Dauer < 10 Jahren ohne weiteren Risikofaktor.

Eine klare Indikation zur Thrombozyten-Aggregationshemmung besteht bei Personen mit klinisch manifester kardiovaskulärer Erkrankung. Diese Patienten sollten Acetylsalicylsäure (75–100 mg täglich), oder, bei dokumentierter Acetylsalicylsäure – Allergie Clopidogrel (75 mg täglich) erhalten.

Für Patienten mit hohem oder sehr hohem Risiko ohne kardiovaskuläre Erkrankung kann eine Thrombozytenaggregationshemmung unter Berücksichtigung der individuellen Situation sowie von Kontraindikationen erwogen werden.

Für Patienten mit mittlerem Risiko ist eine Thrombozytenaggregationshemmung nicht empfohlen.

## Blutungskomplikationen

Grundsätzlich muss dem möglichen Nutzen der Therapie die Rate an Blutungskomplikationen gegenübergestellt werden. Diese liegen für den hämorrhagischen Insult bei 1/10.000 Patientenjahren und für gastrointestinale Blutungen bei 5/1000 Patientenjahren; das Blutungsrisiko unter Acetylsalicylsäure ist stark altersabhängig.

### Nutzen-Risikoabwägung

Bei einem Risiko für schwere kardiovaskuläre Ereignisse von 10–20/10 Jahre entspricht die Zahl verhinderter schwerer kardiovaskulärer Ereignisse in etwa der Zahl verursachter Blutungen. Daher kann erst ab einer kardiovaskulären Ereignisrate von > 2 % eine Reduktion kardiovaskulärer Ereignisse erwartet werden die größer ist als die Steigerung der Blutungskomplikationen.

## Antazide Therapie

In Anlehnung an den Konsensus der Österreichischen Gesellschaft für Gastroenterologie und Hepatologie ist bei folgenden Risikogruppen unter Therapie mit Thrombozytenaggregationshemmern der Einsatz einer antaziden Therapie indiziert:

1. Alter > 65 Jahre
2. Gastrointestinale Ulkusanamnese
3. Begleittherapie mit NSAR, Cortison, Antikoagulanzien, anderen Thrombozytenaggregationshemmern

## Evidenzlage

Eine Meta-Analyse der ATTC aus dem Jahr 2002 zeigt, dass Patienten mit Diabetes bei erhöhtem Risiko im gleichen Ausmaß von niedrig dosierter Acetylsalicylsäure profitieren wie Patienten ohne Diabetes ([2], Evidenzklasse A).

Generell legt die Literatur in der Primärprävention geschlechtsspezifische Effekte von Acetylsalicylsäure nahe. Bei Männern scheint eher der Myokardinfarkt, bei Frauen eher der Schlaganfall in seiner Häufigkeit beeinflusst zu werden ([3]; Evidenzklasse A).

Zwei Studien an Patienten mit Typ 2 Diabetes [4, 5] konnten zwar keinen signifikanten Effekt für Acetylsalicylsäure in der Primärprävention zeigen, müssen jedoch auf Grund der Studiengröße als underpowered angesehen werden.

Eine große Meta-analyse (auf Basis individueller Einzeldaten) bestätigt ältere Analysen [6] dahingehend, dass Hochrisiko-Patienten mit Diabetes in der Primärprävention im gleichen Ausmaß profitieren wie Patienten ohne Diabetes (Evidenzklasse A).

Eine rezente große Studie mit über 15.000 Teilnehmern [7] fand zwar einerseits eine signifikante Reduktion schwerer kardiovaskulärer Ereignisse durch den Einsatz von Acetylsalicylsäure, andererseits aber ein ebenso signifikant häufigeres Auftreten extrakranieller (in 1. Linie gastrointestinaler) Blutungen. Für 5 verhinderte kardiovaskuläre Ereignisse traten 4 zusätzliche Blutungen in diesem Kollektiv das überwiegend keine antazide Therapie erhielt auf.

Eine Therapie mit bis zu 600 mg Acetylsalicylsäure täglich führt nicht zu einem häufigeren Auftreten von Retina- oder Glaskörperblutungen.

## References

[1] The Task Force for diabetes, pre-diabetes, and cardiovascular diseases oft he ESC and the EASD (2019). ESC Guidelines on diabetes, pre-diabetes, and cardiovascular diseases developed in collaboration with the EASD. Eur Heart J.

[2] The antithrombotic trialists collaboration (2002). Collaborative meta-analysis of randomised trials of antiplatelet therapy for prevention of death, myocardial infarction, and stroke in high risk patients. BMJ.

[3] Berger JS, Roncaglioni MC, Avanzini F, Pangrazzi I, Tognoni G, Brown DL (2006). Aspirin for the primary prevention of cardiovascular events in women and men: a sex-specific meta-analysis of randomized controlled trials. JAMA.

[4] Ogawa H, Nakayama M, Morimoto T, Uemura S, Kanauchi M, Doi N, Jinnouchi H, Sugiyama S, Saito Y (2008). Low-dose aspirin for primary prevention of atherosclerotic events in patients with type 2 diabetes: a randomized controlled trial. JAMA.

[5] Belch J, MacCuish A, Campbell I, Cobbe S, Taylor R, Prescott R, Lee R, Bancroft J, MacEwan S, Shepherd J, Macfarlane P, Morris A, Jung R, Kelly C, Connacher A, Peden N, Jamieson A, Matthews D, Leese G, McKnight J, O’Brien I, Semple C, Petrie J, Gordon D, Pringle S, MacWalter R, Prevention of Progression of Arterial Disease and Diabetes Study Group, Diabetes Registry Group, Royal College of Physicians Edinburgh (2008). The prevention of progression of arterial disease and diabetes (POPADAD) trial: factorial randomised placebo controlled trial of aspirin and antioxidants in patients with diabetes and asymptomatic peripheral arterial disease. BMJ.

[6] The antithrombotic trialists collaboration (2009). Aspirin in the primary and secondary prevention of vascular disease: collaborative meta-analysis of individual participant data from randomised trials. Lancet.

[7] The ASCEND study collaborative group (2018). Effects of aspirin for primary prevention in persons with diabetes mellitus. N Engl J Med.

